# Successful non-native speech perception is linked to frequency following response phase consistency

**DOI:** 10.1016/j.cortex.2017.05.005

**Published:** 2017-08

**Authors:** Akihiro Omote, Kyle Jasmin, Adam Tierney

**Affiliations:** Department of Psychological Sciences, Birkbeck, University of London, London, United Kingdom

**Keywords:** Auditory, English, FFR, Japanese, Speech

## Abstract

Some people who attempt to learn a second language in adulthood meet with greater success than others. The causes driving these individual differences in second language learning skill continue to be debated. In particular, it remains controversial whether robust auditory perception can provide an advantage for non-native speech perception. Here, we tested English speech perception in native Japanese speakers through the use of frequency following responses, the evoked gamma band response, and behavioral measurements. Participants whose neural responses featured less timing jitter from trial to trial performed better on perception of English consonants than participants with more variable neural timing. Moreover, this neural metric predicted consonant perception to a greater extent than did age of arrival and length of residence in the UK, and neural jitter predicted independent variance in consonant perception after these demographic variables were accounted for. Thus, difficulties with auditory perception may be one source of problems learning second languages in adulthood.

## 1 Introduction

Speaking and understanding a second language is a vital skill in an increasingly globalized world. However, learning a second language poses difficulties that surpass those experienced in learning a first language. Native Japanese speakers, for example, struggle to discriminate English /l/ and /r/ (Goto, 1971, Miyawaki et al., 1975). Nevertheless, the difficulties which non-native speech perception presents can be overcome. Native Japanese speakers, for example, through experience (Flege et al., 1995, Ingvalson et al., 2011, MacKain et al., 1981) and training (Bradlow et al., 1999, Bradlow et al., 1996, Iverson et al., 2005, Lim and Holt, 2011, Lively et al., 1994, Lively et al., 1993, Logan et al., 1990, McCandliss et al., 2002) can learn to perceive and produce the distinction between /l/ and /r/ with near-native accuracy. However, there are large individual differences in the degree to which non-native speech sound categories can be successfully acquired: some people achieve approximately native perception and production, while others produce heavily accented speech and struggle to perceive non-native speech even after extensive training (Golestani and Zatorre, 2009, Hanulíková et al., 2012, Kempe et al., 2012, Kempe et al., 2015, Perrachione et al., 2011, Wong and Perrachione, 2007). Understanding the source of these individual differences would be an important step towards the development of tools to boost non-native speech perception.

Learning a non-native speech sound category requires highly precise perception of durational, pitch, and spectral information. One possible source of difficulties with non-native speech perception, therefore, is imprecise auditory perception. Supporting this theory, individual differences in non-native speech perception have been linked to non-verbal auditory perception skills, including amplitude envelope discrimination (Kempe et al., 2012), frequency discrimination (Lengeris & Hazan, 2010), pitch perception (Perrachione et al., 2011, Wong and Perrachione, 2007), and spectral discrimination (Kempe, Bublitz, & Brooks, 2015). However, electrophysiology research has supported a speech-specific source for non-native speech perception difficulties. Díaz et al., 2008, Díaz et al., 2015, for example, found that non-native speech perception ability was linked to neural discrimination of speech sounds but not non-verbal sounds differing in duration or frequency. This link between speech sound discrimination and individual differences in non-native speech perception has been replicated across languages (Garcia-Sierra et al., 2011, Jakoby et al., 2011, Zhang et al., 2009).

Here we examine the link between non-native speech sound perception and auditory processing in Japanese adults learning English as a second language using frequency-following responses (FFRs), an electrophysiological response which reproduces the frequencies present in the evoking sound and reflects early auditory processing in the brainstem and cortex (Coffey, Herholz, Chepesiuk, Baillet, & Zatorre, 2016). The FFR features high test-retest reliability (Hornickel, Knowles, & Kraus, 2012) and reflects neural origins in the brainstem and cortex (Coffey et al., 2016), making it an excellent measure of the robustness of early auditory processing. The precision of FFRs has been linked to individual differences in the development of language skills in children (Hornickel and Kraus, 2013, White-Schwoch et al., 2015), but it remains unknown how FFR precision relates to second language acquisition. Recently, Krizman, Marian, Shook, Skoe, and Kraus (2012) reported that bilingual FFRs more robustly encoded the fundamental frequency (F0) of synthesized speech. Here, therefore, we predicted that non-native speech perception ability would relate to F0 phase-locking. Given that impaired gamma-rate phase-locking has also been shown to characterize children with language impairment (Heim, Friedman, Keil, & Benasich, 2011), we additionally investigated relationships between gamma phase-locking and non-native speech perception.

## 2 Methods

### 2.1 Participants

Participants were 25 native Japanese speakers [13 female, aged 19 to 35 (*M* = 29.3, SD = 4.5)] with English learning experience at secondary school level or above in Japan. Participants were required to have arrived in the UK after the age of 18 and to have been resident there for at least 1 month at the time of testing. Secondary inclusion criteria included normal audiometric thresholds (≤25 dB HL for octaves from 250 to 8000 Hz) and lack of diagnosis of a language impairment. Participants received a mean (SD) score of 7.6 (4.1) on the Musical Experience portion of the Goldsmiths Musical Sophistication Index (Müllensiefen, Gingras, Stewart, & Musil, 2014), indicating low levels of musical training. Mean age of arrival in the UK was 27.8 (4.9) years, and mean duration of residence in the UK was 2.6 (3.1) years. The Ethics Committee in the Department of Psychological Sciences at Birkbeck, University of London approved all experimental procedures. Informed consent was obtained from all participants. Participants were compensated £14 for their participation.

### 2.2 Behavioral measures

English speech perception was tested using the Receptive Phonology Test (Slevc & Miyake, 2006). Each question in this test is designed to assess a phonological contrast in English with which Japanese subjects have difficulty. The test contains three main sections. In the *word* sub-test, participants see a list of 26 word pairs which differ in a single speech sound (e.g., “late/rate”). Participants then hear a list of words and are asked to indicate which of the two words they heard. In the *sentence* sub-test, participants see a list of 25 sentences, with one of the words replaced with a word differing in a single speech sound (e.g., “My sister loves to play with crowns/clowns.”) Participants then hear a list of sentences and are asked to circle the word that they heard. Finally, participants listen to a short story and are given a written version of the story that includes 42 underlined words. Participants are asked to circle any of the underlined words that are mispronounced.

Because the original version of the Receptive Phonology Test featured a speaker of American English, test materials were re-recorded by a native speaker of British English (Received Pronunciation) in soundproof room with a RODE NT1-A Condenser Microphone. Three of the items from the original test were removed, as they feature speech sound contrasts which do not exist in British Received Pronunciation. Audio recordings were presented to participants using Sennheiser HD 25-1-II headphones. See Table 1 for a list of all of the speech sound contrasts included in the test.

**Table 1:** Speech sound contrasts included in the receptive phonology test.

Speech sound contrast	Number of items
**consonants**	**38**
b-v	4
f-h	6
l-r	14
n-ŋ	3
s-ʃ	3
s-θ	8
**vowels**	**32**
æ-ɛ	4
æ-ʌ	6
ɑː-ʌ	1
ɒ-ʊ	1
ɒ-ʌ	2
ʊ-ɔː	5
ɜː-ɑː	5
iː-ɪ	4
ɪ-ɛ	4

### 2.3 Electrophysiology

#### 2.3.1 Stimuli

**Fig. 1:**
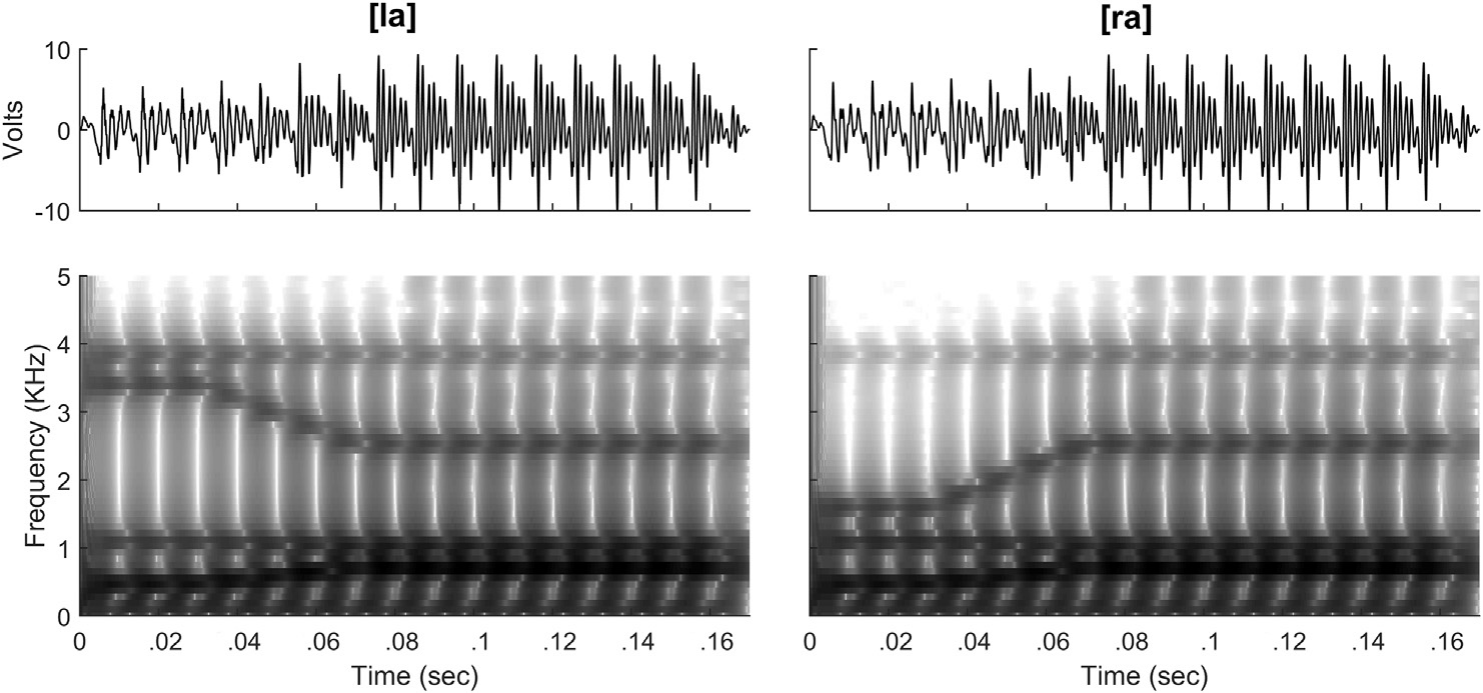
Waveforms (top) and spectrograms (bottom) of synthesized speech stimuli. The [la] and [ra] stimuli differed only in the first 70 msec, and were identical thereafter.

Participants were presented with two 170-msec synthesized speech sounds [la] and [ra]. These syllables were synthesized using a Klatt synthesizer, as implemented in Praat (Boersma & Weenink, 2016). The two syllables differed only during the first 70 msec, during which each had a unique frequency trajectory for the third formant (F3). For [la], F3 was steady at 3400 Hz from 0 to 30 msec, then decreased linearly to 2530 Hz by 70 msec. For [ra], F3 was steady at 1601 Hz from 0 to 30 msec, then increased to 2530 Hz by 70 msec. All other stimulus characteristics were identical across stimuli. F1 was steady at 478 Hz from 0 to 30 msec then increased to 705 Hz by 70 msec. F2 was steady at 1088 Hz from 0 to 30 msec then decreased to 1035 Hz by 70 msec. From 70 to 170 msec F1, F2, and F3 were steady at 705, 1035, and 2530 Hz, respectively. F0 and F4 were constant throughout the stimulus at 100 Hz and 3850 Hz. A cosine off ramp with a duration of 20 msec was used to avoid transients. Fig. 1 displays waveforms and spectrograms for the two stimuli.

#### 2.3.2 Recording parameters

During electrophysiological testing participants sat in a comfortable chair in a soundproof booth with negligible ambient noise and read a book of their choice. Stimuli were presented through Etymotic earphones in alternating polarity at 80 ± 1 dB SPL to both ears with an inter-onset interval of 251 msec. 6300 trials were collected for each stimulus, and stimuli were presented in blocks (i.e., all [ra] trials were collected in a single block). Electrophysiological data were recorded in LabView 2.0 (National Instruments, Austin, TX) using a BioSEMI Active2 system via the ActiABR module with a sample rate of 16,384 Hz and an online bandpass filter (100–3000 Hz, 20 dB/decade). The active electrode was placed at Cz, the grounding electrodes CMS and DRL were placed on the forehead at FP1 and FP2, and the reference electrodes were placed on the earlobes. Earlobe references were not electrically linked during data collection. Offset voltage for all electrodes was kept below 50 mV.

#### 2.3.3 Data reduction

Electrophysiological data reduction was conducted in Matlab R2016a. Offline amplification was applied in the frequency domain for 3 decades below 100 Hz with a 20 dB rolloff per decade. The data was organized into epochs 40 msec before through 210 msec after the onset of the stimulus and baseline corrected. To ensure against contamination by electrical noise a second-order IIR notch filter with a Q-factor of 100 was used with center frequencies of 50, 150, 250, 350, 450, and 550 Hz. A bandpass filter (.1–2000 Hz, 12 dB/oct) was then applied to the continuous EEG recording, and epochs exceeding ± 100 μV were rejected as artifacts. The first 2,500 artifact-free responses to each stimulus polarity then were selected for further analysis.

#### 2.3.4 Data analysis (>70 Hz)

To investigate the precision of neural sound encoding we calculated inter-trial phase-locking. This measure involves calculating the phase consistency at a particular frequency across trials and, therefore, no averaging is necessary. This procedure provides information similar to spectral analysis of average waveforms, but with a higher signal-to-noise ratio and less susceptibility to artifact (Zhu, Bharadwaj, Xia, & Shinn-Cunningham, 2013).

All electrophysiological data analysis was conducted in Matlab 2016a. Parameters for FFR analysis were used for frequencies >70 Hz, in accordance with the standards of previous research on speech FFRs (Bidelman and Krishnan, 2009, Parbery-Clark et al., 2009). For FFR analysis (>70 Hz), phase-locking was calculated within 40-ms windows that were applied repeatedly across the epoch with a 1 msec step size. First, for each trial, a Hanning windowed fast Fourier transform was calculated. Second, for each frequency, the resulting vector was transformed into a unit vector. Third, all of the unit vectors were averaged. The length of the resulting vector—ranging from 0 (no phase consistency) to 1 (perfect phase consistency)—was then calculated as a measure of cross-trial phase consistency. Phase locking factors for [la] and [ra] were averaged together to form a global estimate of an individual's inter-trial phase locking.

This time-frequency data was then averaged in the following manner. First, data were collapsed across the entire response (10–170 msec). Phase-locking at the fundamental frequency (100 Hz) and the second through sixth harmonics was measured by extracting the maximum phase-locking value in a 40-Hz bin centered on each frequency. (Harmonics above 600 Hz were not consistently represented in every single participant and were therefore excluded.) Phase-locking at the harmonics was averaged together to form a general measurement of harmonic encoding. In addition, phase-locking was measured separately in the response to the consonant (10–80 msec) and the response to the vowel (80–170 msec).

#### 2.3.5 Data analysis (<70 Hz)

**Fig. 2:**
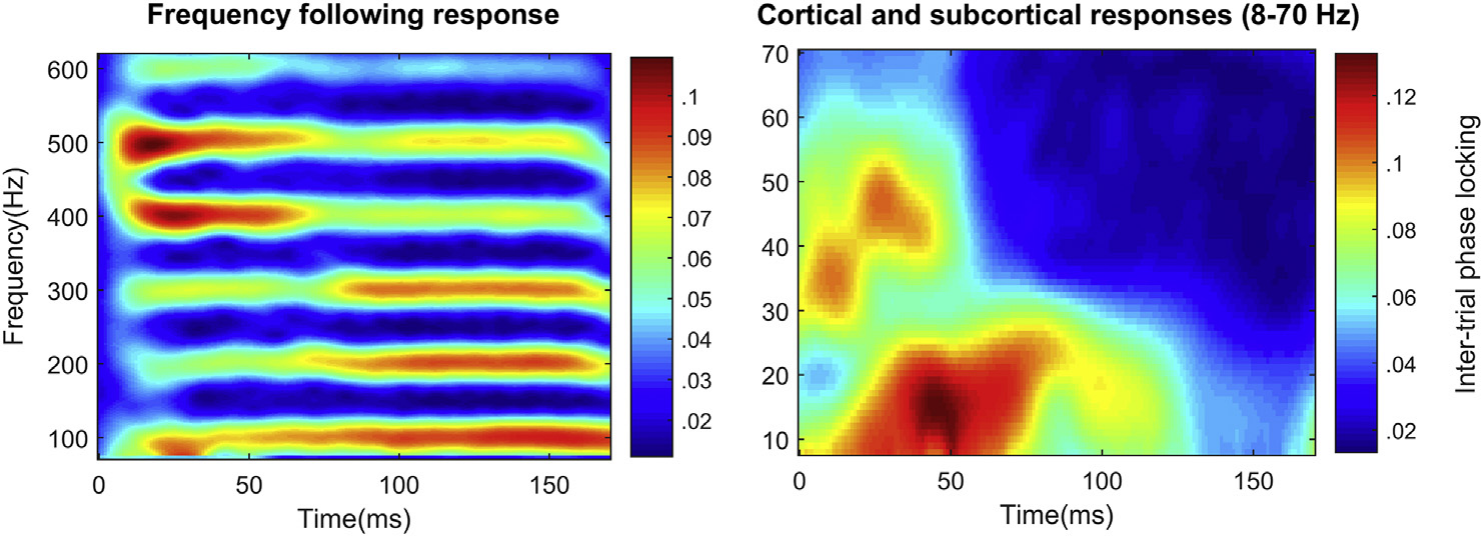
(Left) Time–frequency plot of inter-trial phase locking across all subjects for the frequency following response (71–600 Hz). (Right) Time–frequency plot of inter-trial phase locking across all subjects for the cortical response (8–70 Hz).

For lower-frequency analysis (<70 Hz), phase-locking was calculated within 80-ms windows with a 1 msec step size. Visual inspection of the cross-subject average (see Fig. 2) revealed an increase in phase-locking over baseline between 0 and 60 msec. Gamma phase-locking was quantified, therefore, as the average phase-locking within a window reaching from 0 to 60 msec and between 30 and 70 Hz.

#### 2.3.6 Statistical analyses

Linear models of the behavioral and neural data were constructed using the lm() function with the software package ‘R’, and model comparisons were performed with the anova() function. For comparisons of correlations that shared one variable in common (Steiger, 1980), the r.test() function in the ‘psych’ package from ‘R’ was used.

## 3 Results

**Fig. 3:**
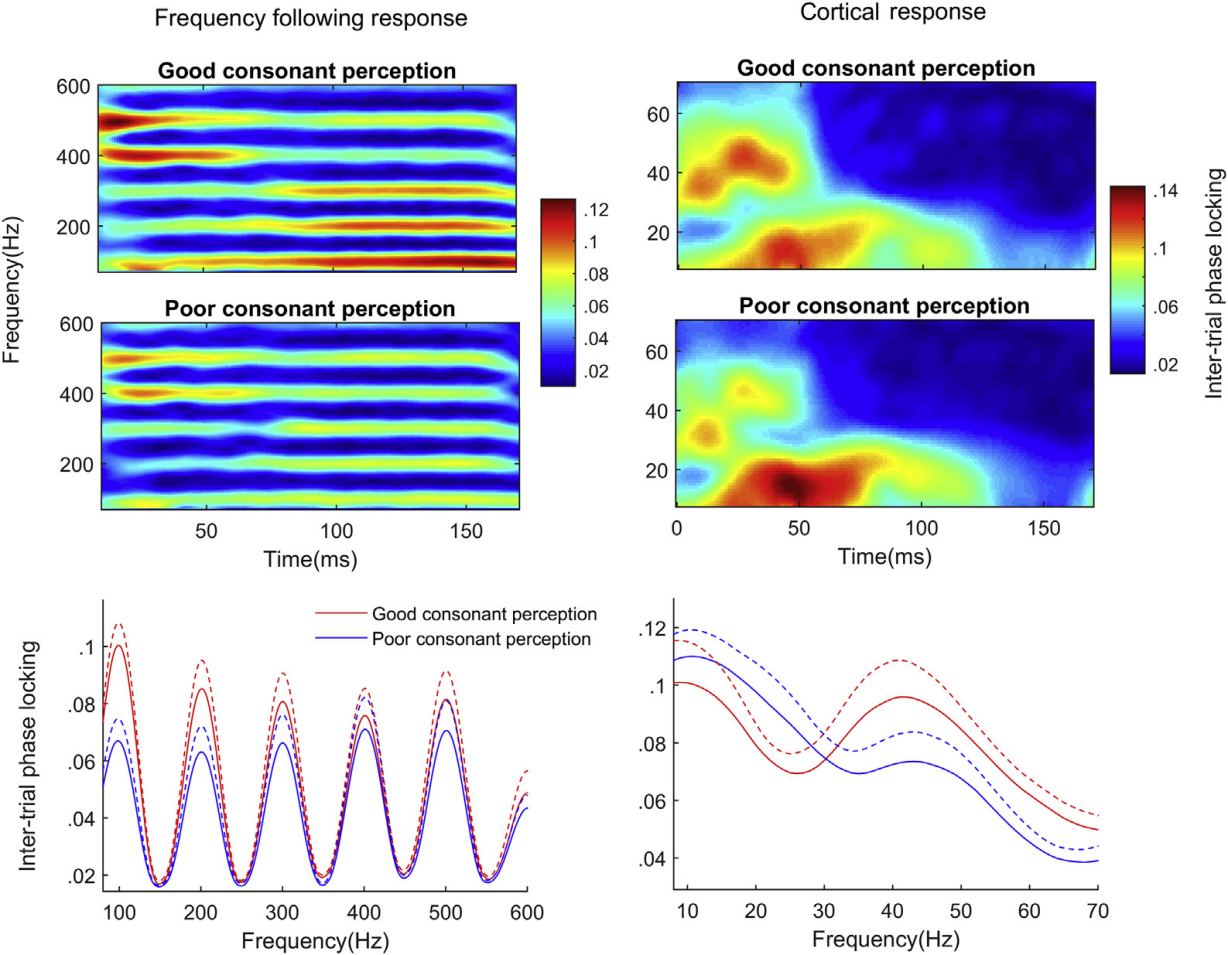
(Left, top) Time–frequency plot of inter-trial phase locking for the frequency following response for participants with good versus poor perception of English consonants. Participants were divided into top and bottom halves based on performance on the consonant portions of the receptive phonology test. (Right, top) Time–frequency plot of inter-trial phase locking for the cortical response for good versus poor consonant perceivers. (Left, bottom) Inter-trial phase locking in the frequency following response as a function of frequency across the entire response (10–170 msec) for good (red) versus poor (blue) consonant perceivers. Error bars are one standard error of the mean. (Right, bottom) Inter-trial phase locking in the frequency following response as a function of frequency across the first 60 msec of the response for good versus poor consonant perceivers.

**Fig. 4:**
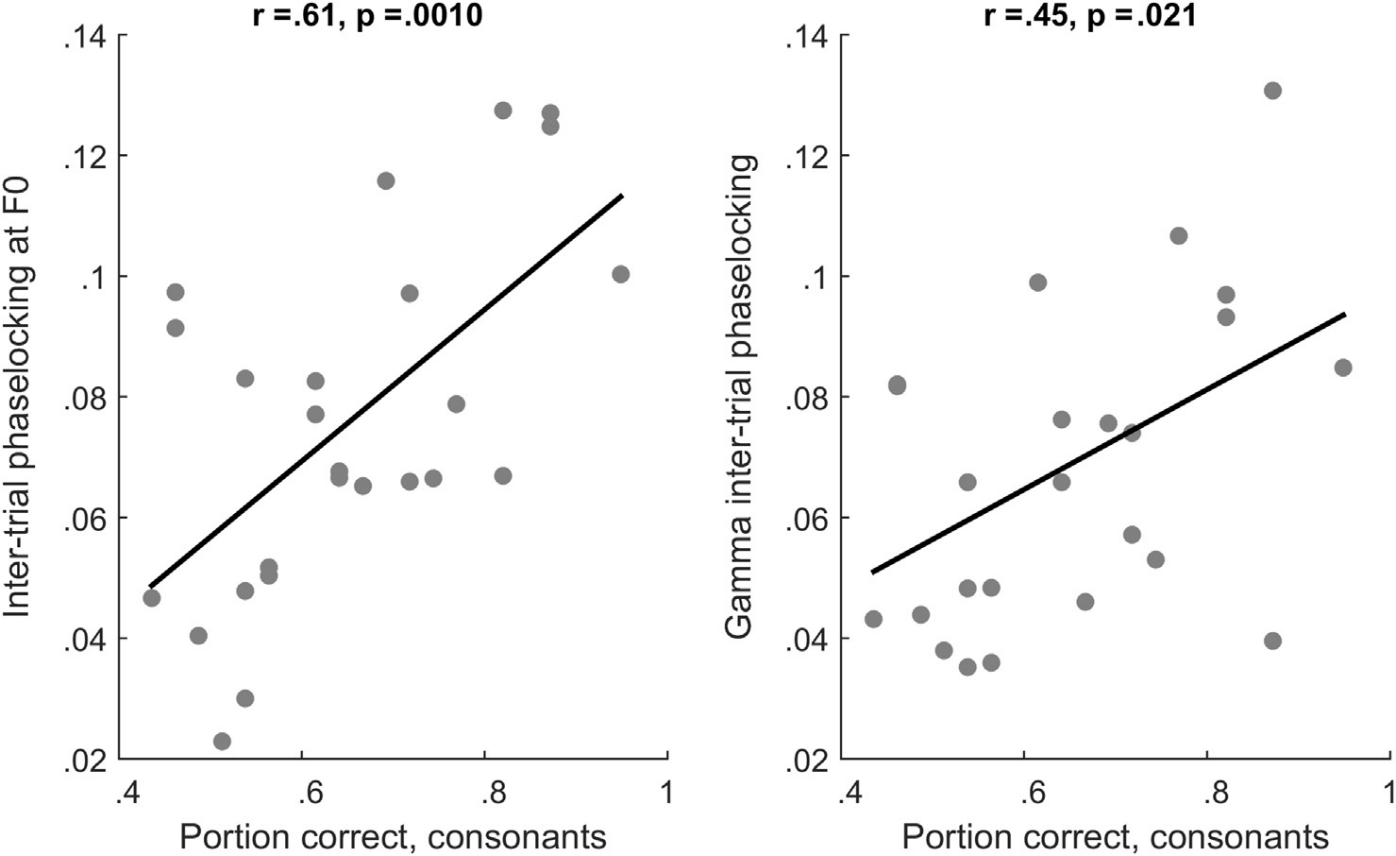
(Left) Scatterplot displaying performance on the consonant portions of the receptive phonology test (displayed as portion correct) versus inter-trial phase locking at the fundamental frequency during the entirety of the frequency following response. (Right) Scatterplot displaying consonant perception versus inter-trial phase locking within the gamma band (31–70 Hz) during the first 60 msec of the cortical response. R-values and *p*-values are derived from Pearson correlations.

First we tested whether the ability to discriminate English consonants was related to our neural measures. Better performance (greater proportion correct items) on the consonant discrimination items of the Phonology Test was associated with greater phase-locking to F0 [*R*^2^ = .379, *F*(1,23) = 14.03, *p* = .001] and with greater phase-locking within the gamma band [*R*^2^ = .21, *F*(1,23) = 6.11, *p* = .021]. Vowel errors were not associated with F0 phase locking [*R*^2^ = .053, *F*(1,23) = 1.30, *p* = .27] or gamma phase locking (*R*^2^ = .000), and phase-locking to the harmonics (H2—H6) did not correlate with performance on consonant items [*R*^2^ = .03, *F*(1,23) = .78, *p* = .34] or vowel items [*R*^2^ = .025, *F*(1,23) = .059, *p* = .45]. The correlation between phase-locking at F0 and consonant perception was significantly greater than the correlation with vowel perception (*T* = 2.76, *p* = .011); similarly, the correlation between gamma phase-locking and consonant performance was significantly greater than the correlation with vowel perception (*T* = 2.95, *p* = .007). The correlation between consonant perception and phase-locking at F0 was significantly greater than the correlation with phase-locking at the higher harmonics (*T* = 2.81, *p* = .01). Fig. 2 displays phase-locking for the cortical evoked response and FFR across all subjects. Fig. 3 displays cortical and FFR phase-locking for good and poor perceivers of English consonants (top-bottom split). Fig. 4 is a scatterplot displaying FFR phase-locking and cortical phase-locking versus consonant perception performance.

One possible explanation for this relationship between English speech perception and F0 phase-locking is that greater familiarity with English speech leads to enhanced encoding of neural responses to English speech sounds. If so, one would expect the relationship between English consonant perception and F0 phase-locking to be limited to the response to the consonant, which did not overlap with any Japanese speech sound. On the other hand, if our results reflect a more general relationship between precise auditory encoding and non-native speech perception, then English consonant perception should also relate to F0-phase-locking in the response to the vowel, which contained formant frequencies appropriate for a Japanese [a] (Nishi, Strange, Akahane-Yamada, Kubo, & Trent-Brown, 2008). We found that F0 phase-locking in the response to the consonant (10–80 msec) correlated with performance on consonant items (*R*^2^ = .426, *p* = .001). F0 phase-locking in the response to the vowel (80–170) also correlated with performance on consonant items (*R*^2^ = .260, *p* = .009). Moreover, the relationship between consonant perception and F0 phase-locking did not significantly differ between these two portions of the response (*T* = .97, *p* = .34).

To further test whether confounding effects of language experience could explain our results, “Age Arrived in UK” and “Years in UK” were used to assess the extent of participants' experience with English. “Years in UK” was cube root-transformed to bring its distribution closer to normality (Shapiro–Wilk *W* = .89, *p* > .01 after transformation). Subjects who were older when they arrived in the UK made more consonant errors, although the correlation was only marginally significant [*R*^2^ = .15, *F*(1,23) = 4.02, *p* = .057]. Age Arrived in UK also correlated negatively with F0 phase locking [*R*^2^ = .17, *F*(1,23) = 4.77, *p* = .039], as well as gamma phase locking [*R*^2^ = .25, *F*(1,23) = 7.71, *p* = .01]. The number of years subjects had spent in the UK prior to testing was correlated with F0 phase locking [*R*^2^ = .31, *F*(1,23) = 7.51, *p* = .004], but not with gamma phase locking [*R*^2^ = .014, *F*(1,23) = .337, *p* = .57].

To assess whether our neural measures predicted variance in phonological competence that could not be simply explained by experience, we fit two linear models: one with age of arrival in the UK and years residence in the UK predicting consonant performance (the “Experience Only” model), and another which also included the consistency of the neural response (F0 phase locking; the “Experience plus Neural model”). The two predictors in the Experience Only model together accounted for 25% of the variance on consonant performance. The Experience plus Neural model with F0 phase locking as a predictor performed significantly better than the Experience Only model [*F*(1,21) = 5.43, *p* = .030], with the F0 phase-locking predictor accounting for an additional 15% of the variance for consonant performance. Including gamma phase locking as an additional predictor only accounted for an additional 1.5% of the variance, and this reduction in error was not significant (*p* = .50).

Finally, to investigate links between individual differences in low-frequency and high-frequency phase-locking, we compared phase-locking in the gamma band to phase-locking in the FFR at F0 and the harmonics. Gamma phase-locking was correlated with phase-locking at both F0 (*R*^2^ = .31, *p* = .004) and the harmonics (*R*^2^ = .17, *p* = .039).

## 4 Discussion

Here we examined English speech perception and neural sound encoding in twenty-five native speakers of Japanese who moved to the United Kingdom as adults. We found that English consonant perception was linked to the degree of phase-locking to the fundamental frequency of the frequency-following response (FFR) to sound and to phase-locking within the gamma band. Vowel perception, however, did not relate to neural phase-locking. The relationship between these neural metrics and English speech perception ability remained significant even after time in the UK and age of arrival were controlled for.

That FFR phase-locking relates to second language speech perception suggests that difficulties with auditory perception can interfere with the acquisition of non-native speech sound categories. On the other hand, we found that non-native vowel perception was not linked to FFR phase-locking, suggesting that vowel perception may depend less on the precision of auditory processing. These findings support previous behavioral research demonstrating relationships between non-native speech perception and auditory abilities including amplitude envelope discrimination (Kempe et al., 2012), frequency discrimination (Lengeris & Hazan, 2010), and spectral discrimination (Kempe et al., 2015). However, language learning is a complex process, and there are likely many ways in which foreign language learning can be disrupted. Only a portion of children with reading impairment, for example, display problems with auditory perception (Ramus et al., 2003), and the causes of adult language learning difficulty are likely to be similarly heterogenous. FFR phase-locking may be a useful metric to help identify people whose difficulties with non-native language perception stem from auditory impairments.

These findings support and extend previous work demonstrating links between the precision of neural sound encoding, language skill, and language experience. Krizman, Slater, Skoe, Marian, and Kraus (2015), for example, found that in Spanish-English bilinguals degree of bilingual experience was linked to the strength of fundamental frequency (F0) encoding in the FFR. Here we replicate this relationship in native speakers of Japanese learning English as a second language, and extend this finding by showing that this same neural metric can also explain individual differences in non-native speech perception, even after language experience is accounted for. Hornickel and Kraus (2013) demonstrated that the inter-trial consistency of the FFR is linked to individual differences in language skills in school-age children; here we show that precise neural encoding of sound is linked to successful adult language learning as well. Chandrasekaran, Kraus, and Wong (2012) showed that the robustness of FFR pitch encoding can predict subsequent short-term learning of lexical tones; here we show that FFR phase-locking is linked to long-term language learning of non-tonal speech sounds.

What is the mechanism underlying this relationship between FFR phase-locking and non-native speech perception ability? One possibility is that FFR phase-locking reflects the precision of temporal perception. FFR phase-locking has been linked to the ability to precisely synchronize movements with sound onsets (Tierney and Kraus, 2013, Tierney and Kraus, 2016 Woodruff Carr, Tierney, White-Schwoch, & Kraus, 2016). This suggests that precise tracking of sound timing relies upon consistent auditory neural timing, as synchronization places stringent demands upon the precision of auditory time perception (on the order of a few milliseconds; Repp, 2000). The ability to track sound timing is also vital for speech perception, as the temporal information contained in the speech envelope contains information relevant to speech sound discrimination (Rosen, 1992); in fact, discrimination of speech sounds is possible even if spectral information is greatly reduced (Shannon, Zeng, Kamath, Wygonski, & Ekelid, 1995). Moreover, non-native speech perception may rely more upon temporal information than does native speech perception. For example, Japanese adults have a strong bias towards the use of temporal information such as closure duration and formant transition duration when distinguishing [la] and [ra], whereas native English speakers rely more heavily upon the frequency of the third formant (Iverson et al., 2005).

We replicate the finding of Krizman et al. (2012) that F0 encoding in the FFR is related to degree of bilingual experience but encoding of the harmonics is not. Moreover, we show that phase-locking at the F0 but not the harmonics is also linked to non-native speech perception ability. The specificity of this relationship was predicted based on these previous findings, but the underlying mechanism remains unclear. One possibility is that this result reflects a relationship between non-native speech perception ability and cortical auditory encoding. There is strong evidence that frequency-following responses at 250 Hz and above are generated within the auditory brainstem, as cooling the inferior colliculus in cats abolishes the scalp-recorded FFR (Smith, Marsh, & Brown, 1975) and patients with inferior colliculus lesions do not display an FFR (Sohmer, Pratt, & Kinarti, 1977). However, both of these studies included no stimuli below 250 Hz, and recent work has suggested that the FFR at 100 Hz is generated within multiple sources, including both cortical and subcortical regions (Coffey et al., 2016). Thus, the higher frequencies of the FFR may reflect a greater contribution from more peripheral areas such as the inferior colliculus, as generally the upper limit of phase-locking to sound is lower in more central structures (Joris, Schreiner, & Rees, 2004). Our finding of a relationship between non-native speech perception ability and phase-locking within both the low-frequency FFR and the gamma band, therefore, may indicate that learning a second language in adulthood relies upon precise cortical but not subcortical auditory processing. This hypothesis cannot be properly evaluated by the current study; however, it could be tested by future work examining FFR phase-locking and non-native speech perception using MEG.

Previous work (Heim et al., 2011, Nagarajan et al., 1999) has demonstrated that children with language learning difficulties have less phase-locked gamma band onset responses to sounds presented with a short inter-stimulus interval (ISI). Here we find that degree of gamma phase-locking is linked to non-native speech perception. Given that our stimuli were presented with a short ISI, this could reflect an impaired ability to process rapidly presented sounds on the part of the participants who struggled to learn to perceive English. Future work could examine this hypothesis by examining links between non-native speech perception and gamma phase-locking to stimuli presented at different ISIs. This enhanced gamma phase-locking in participants better able to perceive English may also reflect greater recruitment of speech processing resources in response to synthesized English speech sounds in these participants, as gamma phase-locking has been shown to be greater for speech stimuli as compared to non-speech stimuli (Palva et al., 2002). This would be consistent with fMRI evidence showing that subjects who are better at learning novel speech sounds display more STG activity when passively listening to speech sounds (Archila-Suerte, Bunta, & Hernandez, 2016). Finally, gamma phase-locking has also been hypothesized to be an important component of speech perception in multi-time resolution models (Poeppel, Idsardi, & van Wassenhove, 2008), in which phonetic information is carried within the gamma band and prosodic information is carried within the delta and theta bands. Greater gamma phase-locking in the participants who were better able to perceive English speech may, therefore, indicate more precise neural encoding of the timing of the speech envelope. This interpretation is supported by our finding that gamma phase-locking was correlated with FFR phase-locking.

One limitation of this work is that it is difficult to rule out the possibility that the link between neural sound encoding and non-native speech perceptual ability is driven by experiential factors. Time spent in the United Kingdom, for example, was linked to both F0 phase-locking and English perception, a relationship which is likely contributing to the link between F0 phase-locking and speech perception performance. However, the relationship between neural sound encoding and non-native speech perception held even after time in the UK and age of arrival were controlled for, suggesting that this relationship partially reflects the dependence of successful non-native language learning on auditory skills. Moreover, the relationship between non-native speech perception and F0 phase-locking held both for the neural response to the consonant, which did not overlap with any Japanese speech sound category, and the response to the vowel, which contained formant frequencies similar to those of the Japanese [a] (Nishi et al., 2008). Nevertheless, in a retrospective study it is difficult to account for all possible confounding experiential factors. This limitation could be addressed in future work in which participants are tested prior to beginning study of a foreign language for the first time or through the use of very short-term training paradigms (Lim & Holt, 2011).
